# Tissue-level implants in the aesthetic zone: why and how?

**DOI:** 10.1038/s41415-026-0084-y

**Published:** 2026-10-09

**Authors:** Ethan Ng, Onur Etöz, Milda Vitosyte, Michael Myint, Shakeel Shahdad, Mital Patel

**Affiliations:** 41415895168001 Centre for Oral Clinical Research, Institute of Dentistry, Faculty of Medicine and Dentistry, Queen Mary University of London, London, UK; Department of Restorative Dentistry, National Dental Centre Singapore, Singapore; 41415895168002 Department of Prosthodontics, Faculty of Dentistry, Mersin University, Mersin, Turkey; Barts and the London School of Medicine and Dentistry, Institute of Dentistry, Queen Mary University of London, London, UK; 41415895168003 Barts and the London School of Medicine and Dentistry, Institute of Dentistry, Queen Mary University of London, London, UK; Institute of Odontology, Faculty of Medicine, Vilnius University, Vilnius, Lithuania; 41415895168004 Barts and the London School of Medicine and Dentistry, Institute of Dentistry, Queen Mary University of London, London, UK; 41415895168005 Barts and the London School of Medicine and Dentistry, Institute of Dentistry, Queen Mary University of London, London, UK; Department of Restorative Dentistry, The Royal London Dental Hospital, Barts Health NHS Trust, London, UK

## Abstract

Tissue-level (TL) implants, originally developed to eliminate the need for a second surgical procedure, are increasingly being used in the aesthetic zone due to their unique design advantages. The vertical platform shift positions the implant-abutment junction coronally, minimising marginal bone loss and optimising the crown-to-implant ratio. The machined, divergent collar may support space maintenance during guided bone regeneration and the buccal soft tissue contour. Additionally, favourable pre-determined emergence angles may be helpful in reducing mechanical and biological complications while enhancing implant-abutment stability. Achieving predictable outcomes requires meticulous prosthetically driven 3D planning, integrated digital workflows, precise surgical execution, and careful handling during the prosthetic phase. This manuscript presents the rationale for using TL implants in the aesthetic zone and offers practical guidance to achieve successful outcomes in clinical practice.

## Key points

- Tissue-level (TL) implants can be placed in the aesthetic zone with excellent results.
- The design of TL implants is associated with several inherent advantages.
- Achieving successful outcomes requires comprehensive pre-operative planning and precise prosthetically driven placement.

## Introduction

The replacement of a missing tooth with an implant-supported crown has become a predictable and increasingly common treatment modality in contemporary clinical practice. However, implant rehabilitation in the aesthetic zone is challenging due to the high aesthetic demands and anatomical limitations associated with this area.^1^ Previous studies have shown that only 10% of anterior teeth have a labial bone thickness >1 mm either at crestal or mid-root level.^2^^,^^3^ Furthermore, most of this bone is composed of bundle bone which gets its blood supply from the periodontal ligament (PDL). Following tooth extraction, loss of the PDL results in resorption of the bundle bone and subsequent dimensional reduction of the buccal and palatal bone walls.^4^ Consequently, unassisted socket healing is associated with substantial alveolar ridge remodelling, with studies reporting up to 63% horizontal bone loss.^5^^,^^6^ Many anterior cases require bone regeneration, soft tissue augmentation, or both procedures to re-establish buccal convexity at implant sites.^7^^,^^8^

**Fig. 1:**
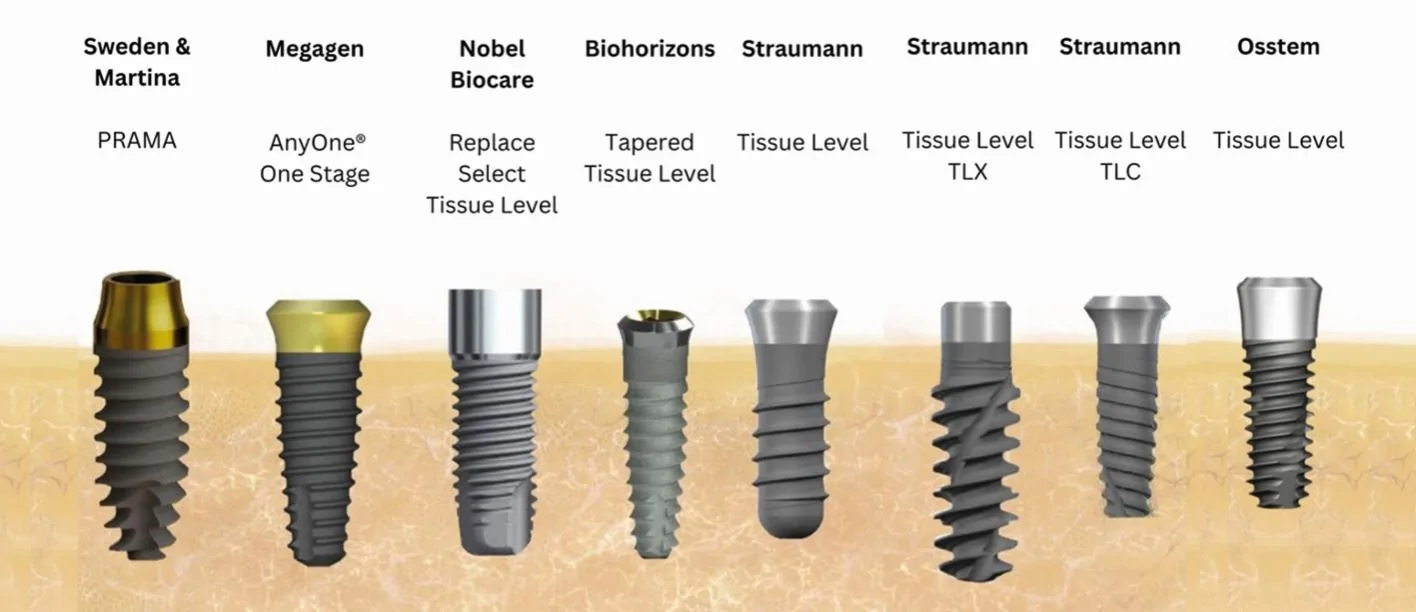
Examples of various tissue-level implant designs

Over the years, dental implants have undergone significant advancements in both materials and treatment protocols. Innovations in implant timing and loading strategies have enabled clinicians to tailor treatments to individual patient needs and their own clinical expertise.^9^^,^^10^ Machined collar implants, also known as polished collar implants or tissue-level (TL) implants, have a machined coronal portion that acts as a transition zone between the rough surface of the implant and the abutment (Fig. 1).

The use of TL implants in the aesthetic zone remains a topic of debate due to challenges such as limited control over the emergence profile^11^ and the perceived higher risk of implant collar exposure.^12^ Despite these concerns, long-term evidence suggests that TL implants in the aesthetic zone can achieve satisfying aesthetic results, in terms of Pink Esthetic Scores, as compared to bone level implants.^13^ Other advantages such as reduced marginal bone resorption^14^ and improved papilla formation^15^ have been reported as well. Furthermore, favourable clinical and radiographic results may be achieved irrespective of the vertical mucosal thickness, emphasising the importance of establishing an appropriate biological peri-implant supracrestal tissue height.^16^ This paper aims to present the rationale for using TL implants in the aesthetic zone and outline strategies for achieving successful outcomes.

## Why use tissue-level implants in the aesthetic zone?

### Vertical platform shift

**Fig. 2:**
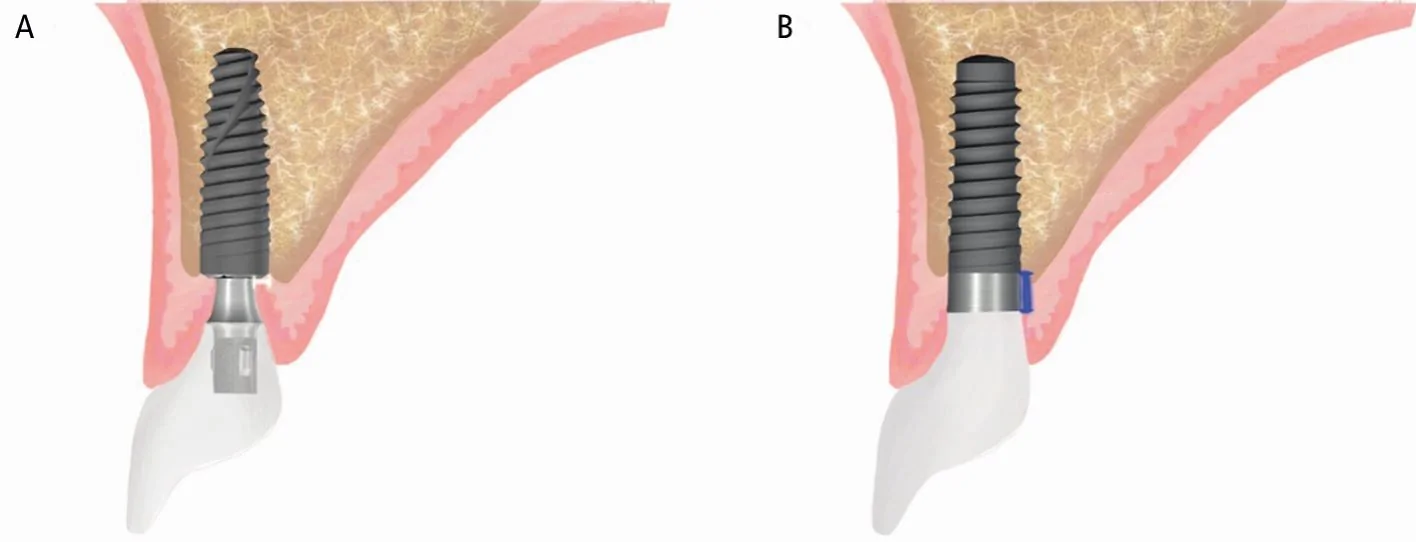
A horizontal platform shift (white line) relocates the inflammatory infiltrate laterally away from the bone (A), while a vertical platform shift (blue line) does the same thing in a vertical direction (B)

The platform-switching concept is based on a horizontal mismatch between the implant and abutment, which moves the implant-abutment junction away from the bone crest by a fraction in the horizontal direction (Fig. 2A). With this, there is a shift in the inflammatory infiltrate away from the crestal bone and facilitates establishment of the biological width.^17^ Horizontal platform-switching has been extensively documented as having a protective effect on implant hard tissue outcomes,^18^ and is a feature of most modern implant macro designs. Conversely, vertical platform-switching, a less understood concept, also involves moving the implant-abutment junction away from the bone crest but in a vertical direction (Fig. 2B), which has also been shown to reduce marginal bone loss.^19^ More recent clinical studies support this finding, demonstrating that abutment height significantly influences bone loss around implants.^20^^,^^21^^,^^22^

Early research showed that the dimensions of the peri-implant soft tissue, or supracrestal complex, are influenced by the position of the microgap (implant-abutment interface) relative to the bone.^23^ In particular, when this interface is positioned further away from the bone, the resulting soft tissue dimensions more closely resemble those of the periodontal supracrestal tissue complex around natural teeth, and are associated with reduced inflammatory changes at the peri-implant margin. Contemporary understanding frames the implant supracrestal complex as a coordinated biological unit comprising the peri-implant mucosa, underlying bone, implant–abutment connection, restorative components, and the adjacent biofilm environment, all of which interact to influence long-term tissue stability and disease susceptibility.^24^

A clinical study evaluating the impact of horizontal mismatch distance and prosthetic abutment height on marginal bone loss found that a greater mismatch did not necessarily result in less bone loss, whereas a taller abutment height was protective.^25^ This suggests that along with a slight horizontal platform shift, taking the crown-abutment junction away vertically from the crest of the bone in bone level implants helps preserve bone levels. For TL implants, both the implant-abutment junction and the crown-abutment junction are located at the level of the coronal machined collar, thereby positioning these interfaces vertically away from the rough implant surface and the crestal bone. The amount of vertical platform shift may vary depending on the tissue-level implant system (Table 1).

**Table 1:** Machined collar heights across commonly used tissue-level implant systems

**Manufacturer**	**System**	**Machined collar height**
Straumann	Tissue Level Standard Plus or Standard	1.8 or 2.8 mm
Nobel Biocare	Replace Select TC	3.0 mm
BioHorizons	Tapered Tissue Level	2.0 mm
MegaGen	AnyOne Onestage	1.8 or 2.2 mm
Sweden & Martina	Prama	1.8, 2.8, or 3.8 mm
Osstem	SS System	1.8 or 2.8 mm

The position of the restorative margin in relation to the implant-supported prosthesis has also been identified as a critical risk indicator for peri-implantitis.^26^ Nine-year data from a large Swedish cohort demonstrated that a distance of ≤1.5 mm between the prosthetic margin and the crestal bone was associated with an increased odds ratio (OR: 2.3) for moderate to severe peri-implantitis.^27^ It is worth noting that in this study, implants with the lowest risk of peri-implantitis were all tissue-level design implants. Therefore, a vertical platform shift may provide similar protective benefits against marginal bone loss as horizontal platform switching. This observation is further supported by clinical studies that found no significant differences in marginal bone loss between platform-switching designs and TL implants.^28^^,^^29^

### Soft tissue seal

Horizontal platform switching creates a horizontal offset between the implant platform and the abutment, which has been histologically associated with a circumferential reorganisation of supracrestal collagen fibres, contributing to the stabilisation of peri-implant soft tissues around the restoration.^30^

**Fig. 3:**
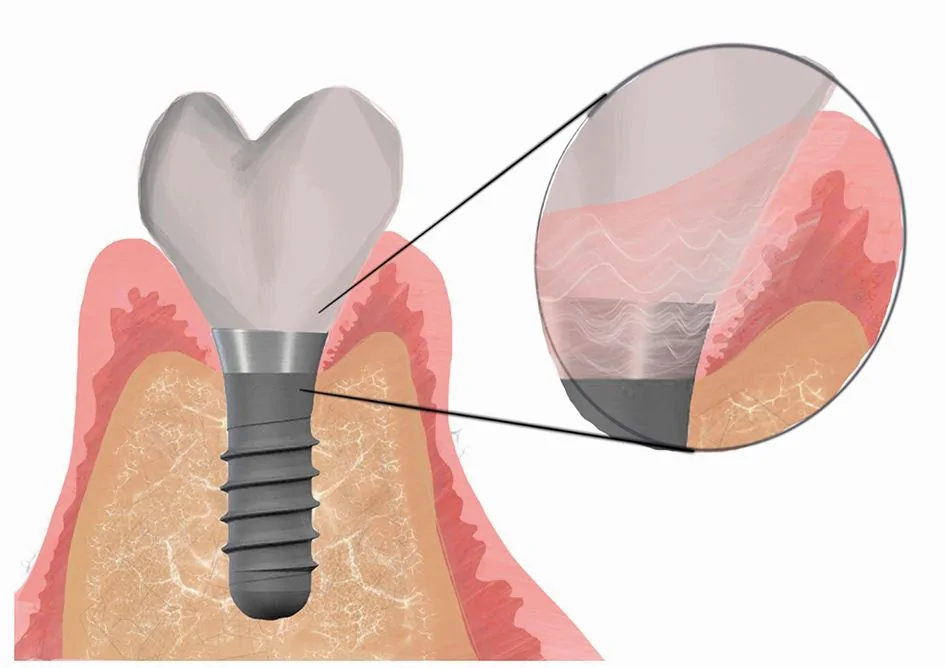
Illustration of a tissue-level implant with healthy peri-implant tissues, showing the supracrestal tissue attachment around the machined collar

On the contrary, vertical platform switching enables simultaneous mucointegration and osseointegration. A key advantage of this design is that the supracrestal tissue established during the primary healing phase remains undisturbed during abutment connection and the prosthetic phase of treatment (Fig. 3). Furthermore, studies investigating the different implant transmucosal surfaces have identified machined titanium and a polished neck area as the most favourable for cell adhesion and connective tissue attachment.^31^^,^^32^

Often during the prosthetic phase, the abutment/crown needs to be removed at multiple appointments to allow for soft tissue moulding. The TL design allows for multiple reconnections without compromising the soft tissue seal. Clinically, this could be significant as some studies have demonstrated that repeated reconnections of healing abutments are associated with significantly greater bone loss compared to the use of a definitive abutment from the outset.^33^^,^^34^

### Divergent space-maintaining collars may support guided bone regeneration

**Fig. 4:**
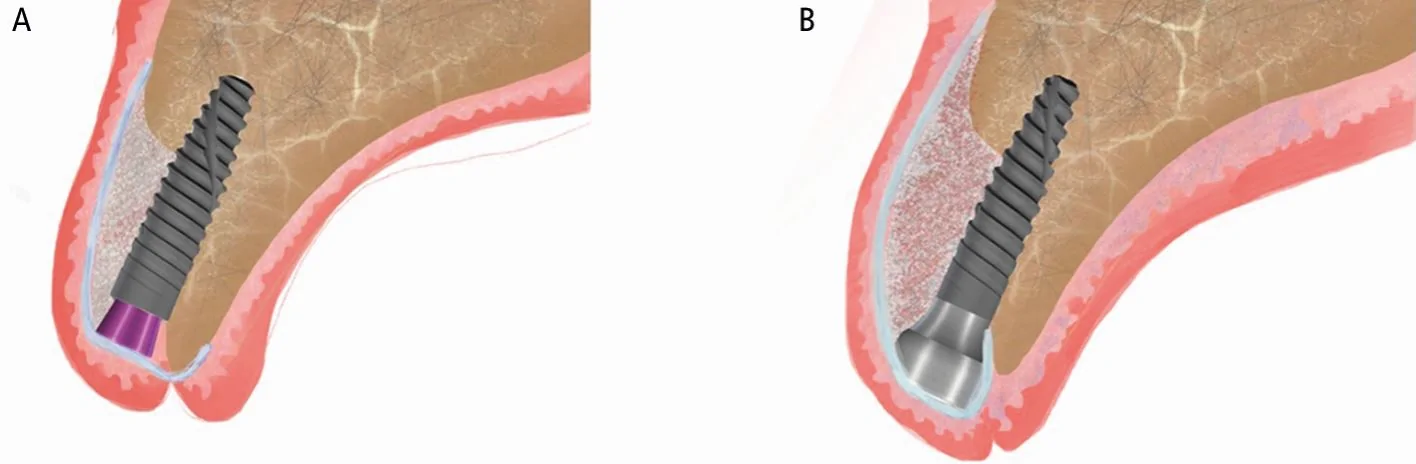
A tenting effect in guided bone regeneration to maintain space and reduce graft displacement can be achieved by using a healing abutment (A) or a tissue-level implant (B)

The influence of implant macro design on the outcomes of simultaneous guided bone regeneration (GBR) has been recently reported.^35^ In this systematic review and meta-analysis, space-maintaining machined collars were found to achieve greater vertical bone gains compared to non-collared implants, attributed to a tenting effect (Fig. 4). Specifically, implants with transmucosal divergent collars demonstrated the greatest reduction in defect height. It is hypothesised that the divergent collar, which is wider than the body of the implant, will minimise soft tissue pressure on the implant neck, thereby preventing displacement of the particulate graft following flap closure – a mechanism akin to using a 2 mm healing abutment instead of a cover screw in BL implants.^36^ Furthermore, TL implants exhibit similar marginal bone level changes to bone level implants even when simultaneous GBR is performed,^37^^,^^38^ and this bone can be maintained coronal to a sand-blasted, large-grit, acid-etched surface.^39^

### Favourable pre-determined emergence angle

Prosthetic factors have been identified as part of the multifactorial pathogenesis of peri-implantitis by predisposing to biofilm accumulation.^40^ Indeed, wider restorative emergence angles have been linked to increased marginal bone loss and compromised integrity of peri-implant supracrestal tissues.^41^ Clinical studies have identified an emergence angle exceeding 30° as a significant risk factor for peri-implantitis.^42^^,^^43^ Notably, this correlation was not observed in TL implants, likely due to the pre-determined emergence angle set by the divergent collar design.

### The TL collar provides biomechanical strength

Prosthetic complications associated with implant-supported prostheses, while generally minor, are relatively common and affect nearly half of all prostheses.^44^ Biomechanically, the implant-abutment interface is recognised as the weakest point in the implant-restorative system. Screw loosening is the most frequent complication, whereas abutment screw fractures and implant fractures are comparatively rare.^45^ Micromovement at the implant-abutment interface has also been implicated in marginal bone loss.^46^

The machined collar of a TL implant can offer a protective effect on the implant-abutment assembly. *In vitro* load testing of three different titanium abutments torqued to TL implants over two million cycles demonstrated no impact on the stability of the implant-abutment unit, nor did it result in any prosthetic complications.^47^ Clinically, this design feature is particularly significant in two scenarios. First, when narrow-diameter implants (3.3 mm) are placed in atrophic ridges, the corresponding abutments of bone-level implants are even narrower, potentially increasing the risk of mechanical complications, including abutment fracture. Second, the design of TL implants may offer biomechanical advantages in cantilever situations, where a single implant supports two anterior restorations because of limited mesiodistal space or to optimise aesthetic outcomes.^48^

### The TL collar improves crown-to-implant ratio

A high crown-to-implant (C/I) ratio may be encountered in clinical situations such as increased interocclusal space or vertical ridge deficiency. From a biomechanical perspective, exceeding a certain C/I threshold can elevate stress concentrations at the bone-implant interface, potentially contributing to crestal bone loss and compromising implant success.^49^ This topic was part of the 5^th^ European Association for Osseointegration Consensus Conference, where a C/I ratio of 0.86–2.14 was identified as the threshold for single, non-splinted implants without a significant increase in biological or technical complications.^50^

**Fig. 5:**
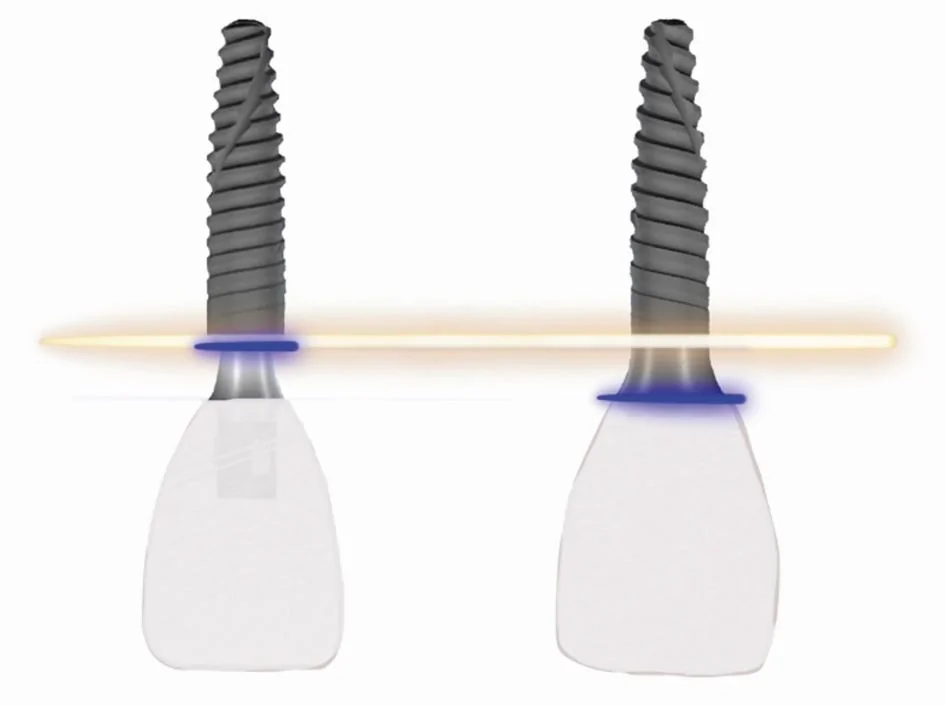
Comparison of crown-to-implant ratios, highlighting how the integrated transmucosal collar of a tissue-level implant (right) reduces this ratio relative to a bone level implant (left)

TL implants may contribute to a more favourable C/I ratio in two ways (Fig. 5). First, the integrated transmucosal collar positions the implant-abutment interface supracrestally, effectively reducing the clinical crown height compared to bone level implants, which require additional abutment height above the bone. Second, the design of TL implants often limits the need for deep subcrestal placement and can compensate for mild vertical bone deficiencies. These features promote a more balanced C/I ratio and enhance load distribution, particularly in scenarios where crown height is excessive or when short implants are used.

Emerging medium- to long-term data support the efficacy of short implants (≤6 mm), demonstrating survival rates comparable to those of longer implants placed with sinus floor elevation or vertical ridge augmentation, while also offering advantages such as reduced treatment time, cost, and patient morbidity.^51^^,^^52^ Interestingly, in a recent clinical study comparing bone level (7 mm length, placed 1 mm subcrestally) and tissue-level (5 or 6.5 mm length) short implants in the posterior maxilla, TL implants demonstrated superior marginal bone stability after one year of functional loading.^53^

### Potentially reduces the need for soft tissue grafting

Correcting the appearance of a resorbed alveolar process can be categorised into minor ridge discrepancies (<2 mm), where provisional restorations may assist in developing an appropriate soft tissue profile, and major discrepancies, which typically require the addition of a connective tissue graft alongside the provisional restoration.^54^ In practice, achieving optimal buccal convexity in the aesthetic zone has been challenging, even after hard tissue augmentation, prompting several authors to advocate for additional soft tissue grafting in certain cases.^7^^,^^8^

**Fig. 6:**
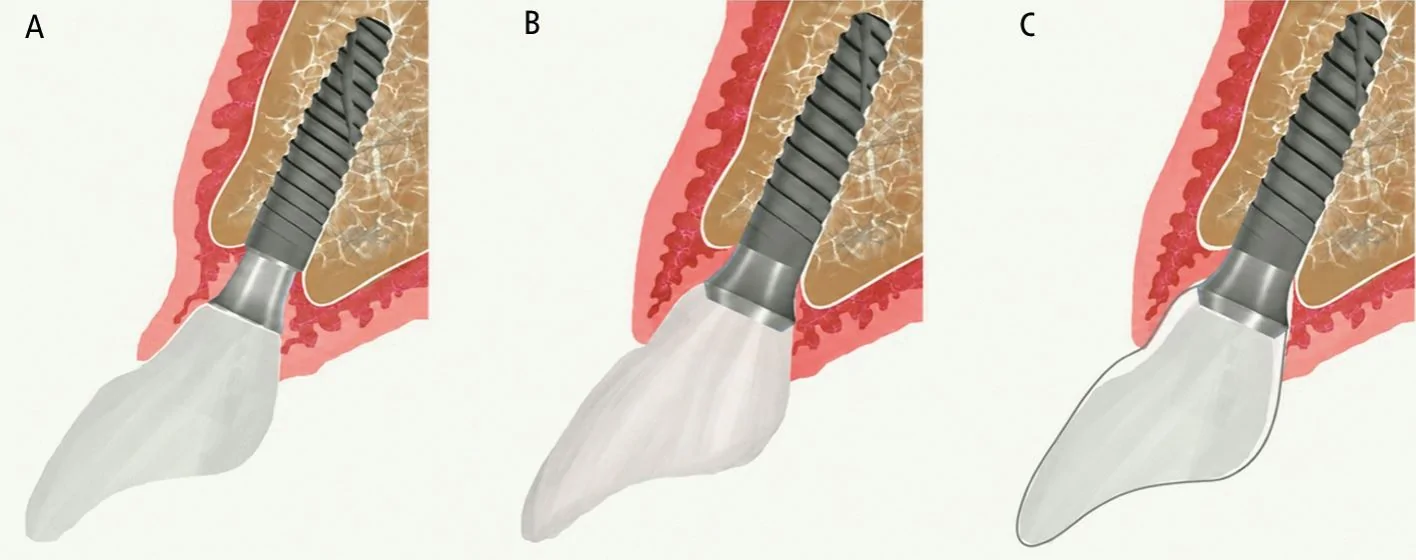
Buccal soft tissue support following restoration with a bone level implant (A) and a tissue-level implant (B). The grey outline illustrates the potential enhancement in crown contour afforded by a tissue-level (TL) collar, which may contribute to improved support of the buccal soft tissue profile (C)

**Fig. 7:**
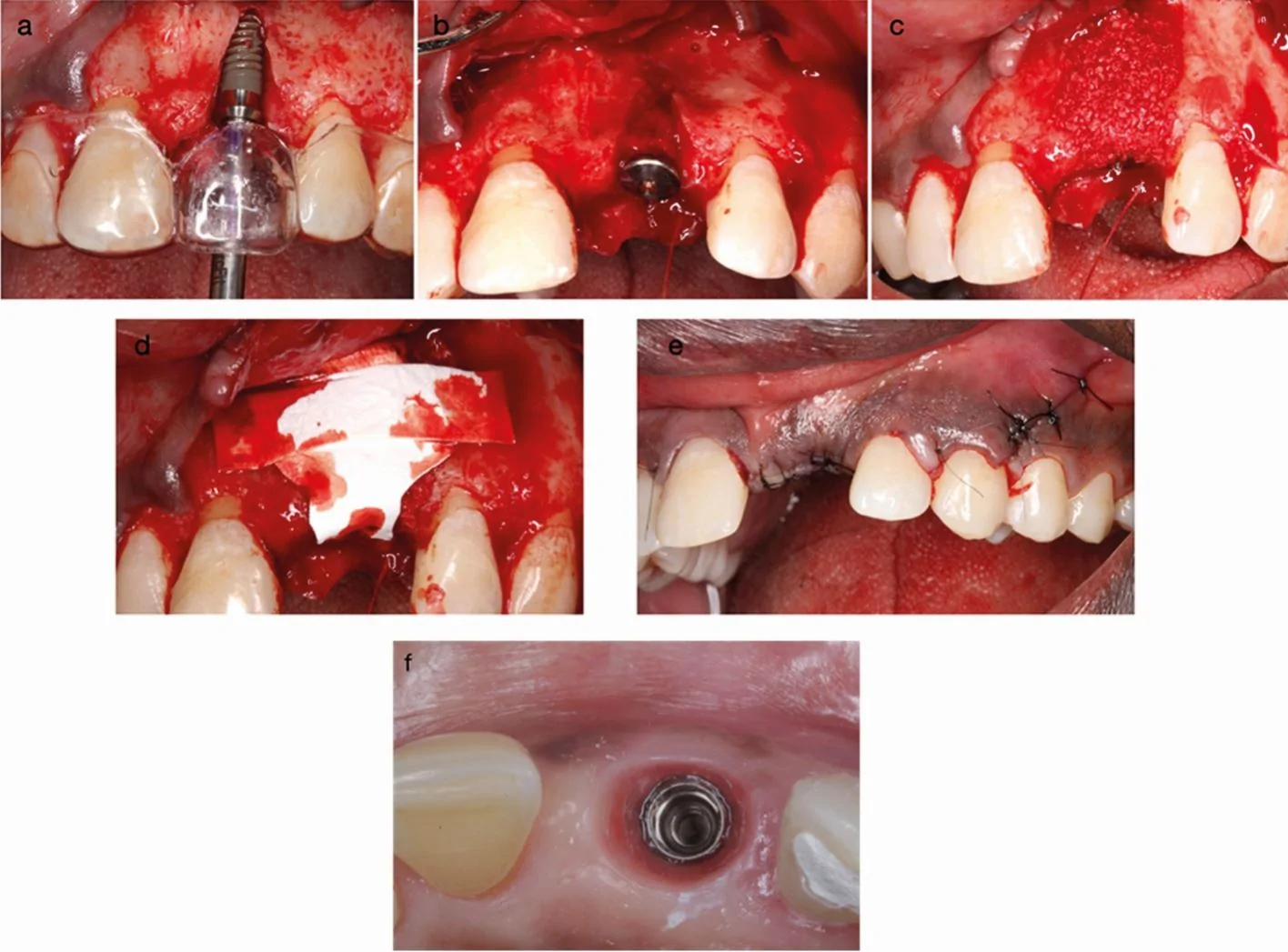
Clinical case demonstrating type 2 implant placement with simultaneous guided bone regeneration. Buccal dehiscence is evident at implant placement (A). Autogenous bone is applied directly to the implant surface (B), followed by layering of bone substitute over the autogenous bone (C). A double membrane technique is used to cover the graft (D), and primary closure is achieved with simple interrupted sutures (E). The final result shows a re-established buccal contour without the use of a soft tissue graft (F)

A limitation of the existing literature is the predominant focus on bone level implants. Based on the author's clinical observations, the utilisation of TL implants appears to reduce the necessity for soft tissue grafting procedures. This may be because the divergent collar of TL implants naturally supports the subcritical contour by giving approximately 2 mm of implant surface that can support bone grafting material, and then, in turn, the soft tissue flap (Fig. 6). This configuration may facilitate maintenance of the buccal soft tissue contour around the implant. Together with simultaneous guided bone regeneration, meticulous soft tissue moulding using provisional restorations is often sufficient to re-establish the buccal contour and soft tissue profile effectively (Fig. 7). In cases where there is slight vertical bone loss, a taller machined collar may be chosen to avoid additional bone grafting procedures.

## How may tissue-level implants be used in the aesthetic zone?

Due to the pre-determined emergence angle of TL implants, their use in the aesthetic zone requires meticulous planning and execution to ensure optimal outcomes.

### Pre-surgical planning

**Fig. 8:**
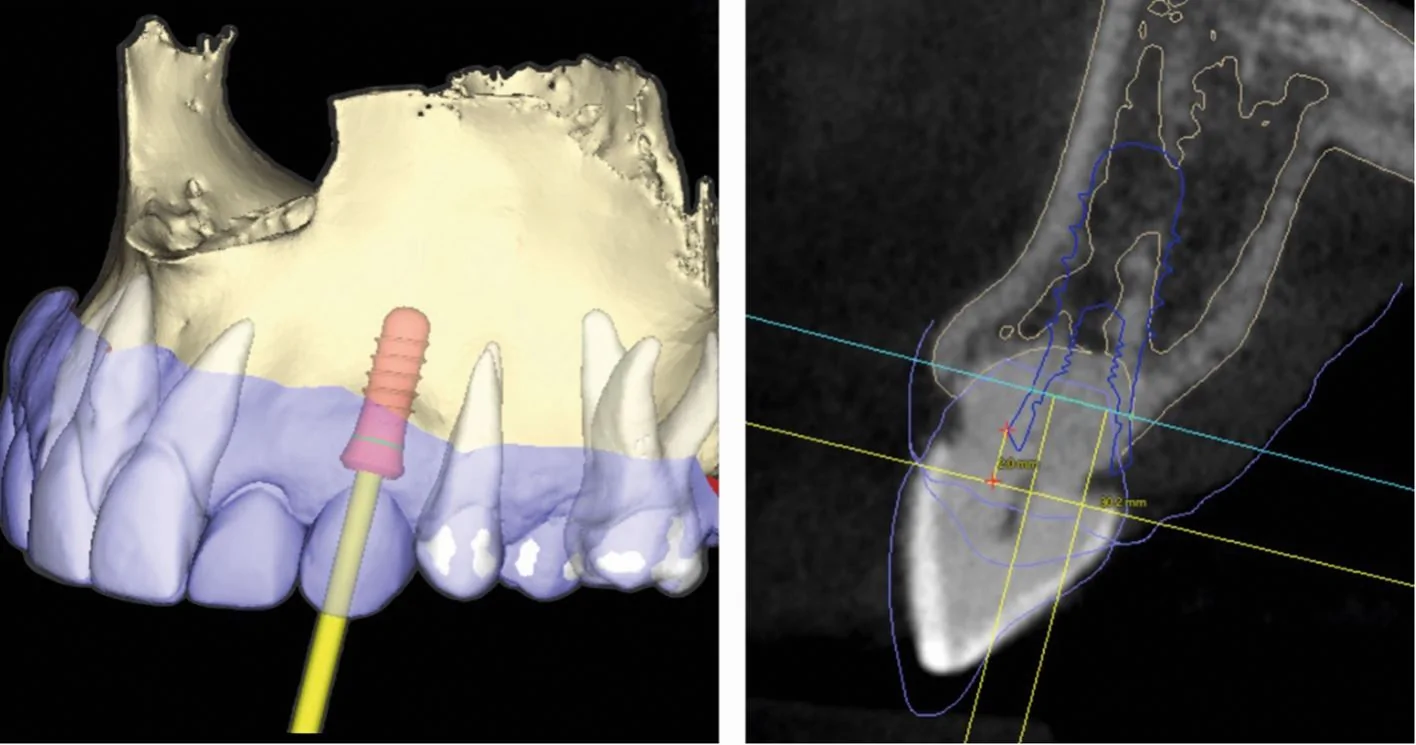
Prosthetically-driven 3D planning and simulated implant placement for guided surgery

The authors use the International Team for Implantology Aesthetic Risk Assessment Tool as the first step in objectively evaluating the complexity and risk profile of each case.^55^ Prosthetically-driven implant positioning is important and dictated by the final restoration's position and the pre-operative anatomical conditions.^56^ Pre-surgical preparation should include an intra-oral scan, digital wax-up and cone beam computed tomography **(**CBCT) imaging to enable virtual planning using relevant software.^57^ Integrating the restorative position with anatomical data from CBCT imaging facilitates precise planning of the 3D implant position, as well as determining the implant length, diameter and platform type (Fig. 8).

Placing implants in the correct 3D position is essential for successful long-term outcomes. Most clinicians aim for palatal screw access holes by placing the implant slightly palatal, and there are well-documented guidelines on the mesio-distal positioning of implants relative to neighbouring teeth or implants.^39^^,^^58^^,^^59^^,^^60^ In contrast, the apico-coronal positioning of implants remains less well understood and is often overlooked in clinical practice. Implants are frequently placed too coronally, resulting in an unfavourable emergence profile of the definitive restoration.

**Fig. 9:**
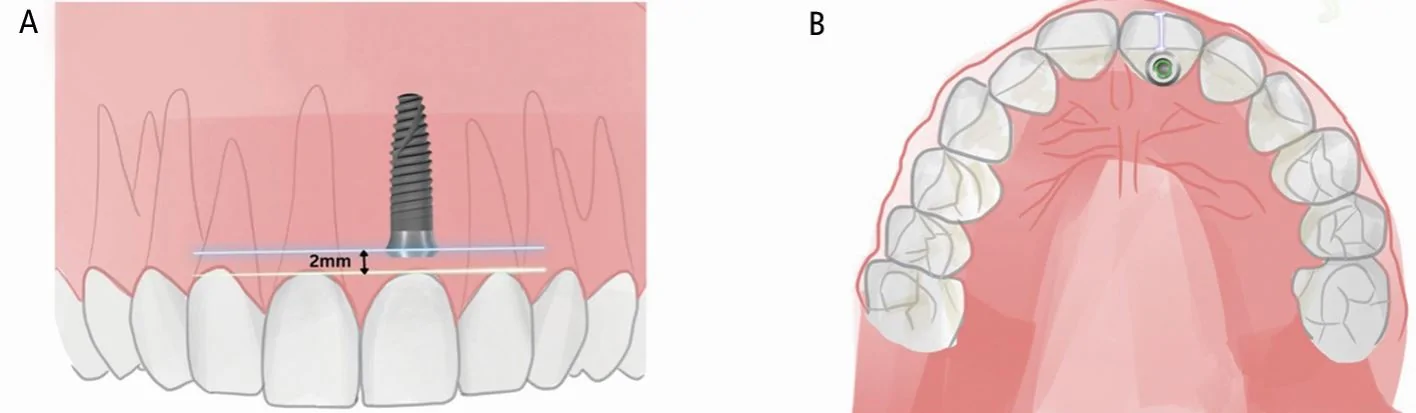
The correct depth of placement for a tissue-level implant is 2 mm from the proposed gingival zenith (A), with the screw access channel emerging from the palatal surface (B)

Regardless of whether a bone-level or tissue-level implant is used, the implant fixture should ideally be positioned in relation to the planned gingival zenith to achieve an optimal restorative outcome. This principle is particularly critical for tissue-level implants, as the position of the transmucosal collar directly influences the emergence profile and peri-implant soft tissue contour. The TL implant fixture should be positioned apicocoronally 2 mm from the proposed mucosal zenith in the aesthetic zone (Fig. 9A), with the screw access channel located on the palatal surface to mitigate the risk of the machined collar becoming exposed (Fig. 9B).

Following meticulous pre-surgical planning, implant placement should prioritise accurate three-dimensional positioning using guided approaches. Fully guided surgery is generally preferred to ensure optimal transfer of the digital plan to the clinical situation, with semi-guided systems providing an intermediate level of control. A vacuum-formed surgical template incorporating clear three-dimensional positioning indicators (e.g., a palatal cut-out extending to the incisal edge and trimming of the stent to the proposed gingival zenith of the tooth to be replaced) may also be used to support verification of the planned implant position.

### Implant surgery

In order to achieve a predictable outcome in the anterior aesthetic zone the authors prefer Type 2 placement, defined as early implant placement following soft tissue healing at 4–8 weeks after tooth extraction.^9^ Type 2 protocol capitalises on reduced osteoclastic activity and the presence of fully matured soft tissue coverage, which may support more stable hard and soft tissue outcomes.^61^ This approach enhances the success of simultaneous bone augmentation to address vertical and horizontal hard tissue deficiencies.^62^^,^^63^

**Fig. 10:**
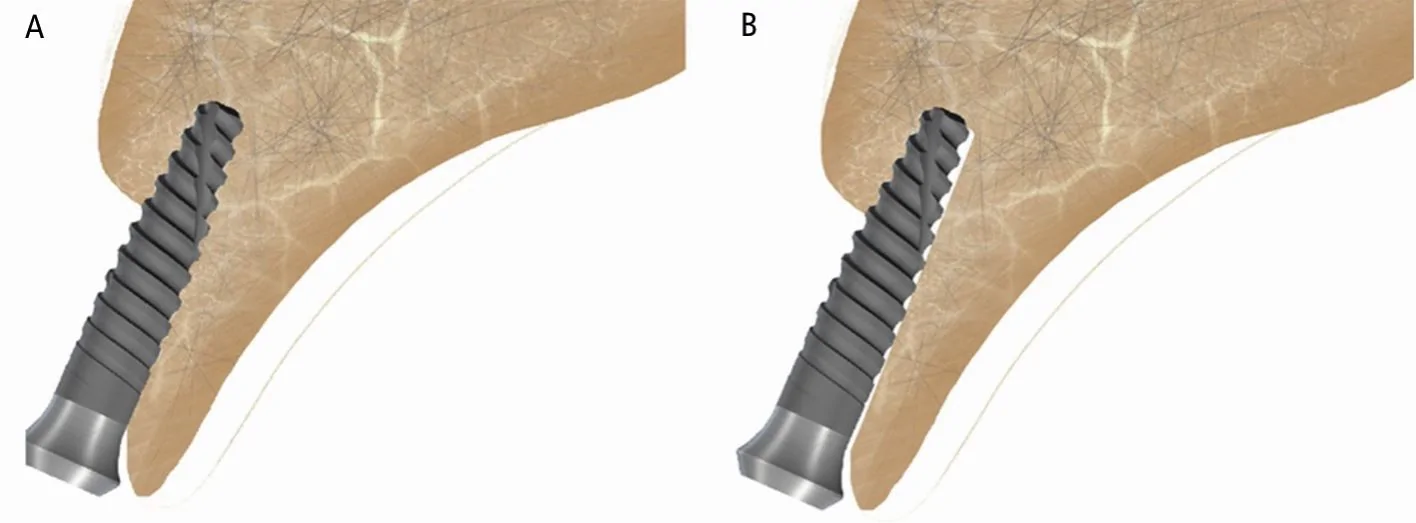
Illustration of the conventional drilling protocol (A) and the modified drilling protocol with overpreparation of the palatal wall (B)

When placing implants in extraction sockets with partial or complete buccal bone deficiency, challenges may arise in achieving the planned implant osteotomy position during seating due to bodily forces pushing the implant toward the vestibular side, with no buccal wall to resist them. To address this, the drilling protocol is modified to include an additional osteotomy step using the final drill to over-prepare the opposite side of the missing wall (palatal wall) slightly to allow passive implant placement (Fig. 10).

In addition to optimal 3D positioning, GBR is essential for reconstructing sufficient hard tissue volume and ensuring stable marginal bone levels. TL implants should not be placed deeper than the optimal depth, as a deeper mucosal tunnel than 3 mm has been associated with a higher incidence of peri-implant mucositis^64^ and also poses a risk of compromised aesthetic outcome.

## Conclusions

The unique design of TL implants renders several advantages that are relevant not just in posterior areas, but in the aesthetic zone as well. While placing these implants is considered challenging in aesthetically demanding areas, this paper highlights the key steps required for successful outcomes and minimises morbidity without compromising results.

## References

[1] Dawson A, Martin W C, Polido W D. *The SAC Classification in Implant Dentistry*. *2nd Ed.* Chicago: Quintessence Publishing, 2022.

[2] Huynh-Ba G, Pjetursson B E, Sanz M *et al*. Analysis of the socket bone wall dimensions in the upper maxilla in relation to immediate implant placement. *Clin Oral Implants Res* 2010; **21:** 37–42. 10.1111/j.1600-0501.2009.01870.x 20070745

[3] Braut V, Bornstein M M, Belser U, Buser D. Thickness of the anterior maxillary facial bone wall – a retrospective radiographic study using cone beam computed tomography. *Int J Periodontics Restor Dent* 2011; **31:** 125–131. 21491011

[4] Araujo M G, Lindhe J. Dimensional ridge alterations following tooth extraction. An experimental study in the dog. *J Clin Periodontol* 2005; **32:** 212–218. 10.1111/j.1600-051X.2005.00642.x 15691354

[5] Tan W L, Wong T L, Wong M C, Lang N P. A systematic review of post-extractional alveolar hard and soft tissue dimensional changes in humans. *Clin Oral Implants Res* 2012; **23(Suppl 5):** 1–21. 10.1111/j.1600-0501.2011.02375.x 22211303

[6] Couso-Queiruga E, Stuhr S, Tattan M, Chambrone L, Avila-Ortiz G. Post-extraction dimensional changes: a systematic review and meta-analysis. *J Clin Periodontol* 2021; **48:** 126–144. 10.1111/jcpe.13390 33067890

[7] De Bruyckere T, Cabeza R G, Eghbali A, Younes F, Cleymaet R, Cosyn J. A randomized controlled study comparing guided bone regeneration with connective tissue graft to reestablish buccal convexity at implant sites: a 1-year volumetric analysis. *Clin Implant Dent Relat Res* 2020; **22:** 468–476. 10.1111/cid.12934 32686234

[8] Wessels R, Vervaeke S, Seyssens L, Eghbali A, Cosyn J. A 5-year cohort study on early implant placement with guided bone regeneration or alveolar ridge preservation with connective tissue graft. *Clin Implant Dent Relat Res* 2020; **22:** 697–705. 10.1111/cid.12948 32939989

[9] Gallucci G O, Hamilton A, Zhou W, Buser D, Chen S. Implant placement and loading protocols in partially edentulous patients: a systematic review. *Clin Oral Implants Res* 2018; **29(Suppl 16):** 106–134. 10.1111/clr.13276 30328194

[10] Morton D, Wismeijer D, Chen S *et al*. Group 5 ITI consensus report: implant placement and loading protocols. *Clin Oral Implants Res* 2023; **34:** 349–356. 10.1111/clr.14137 37750529

[11] Wallner G, Rieder D, Wichmann M G, Heckmann S M. Peri-implant bone loss of tissue-level and bone-level implants in the esthetic zone with gingival biotype analysis. *Int J Oral Maxillofac Implants* 2018; **33:** 1119–1125. 10.11607/jomi.6641 30231100

[12] Menini M, Dellepiane E, Deiana T, Fulcheri E, Pera P, Pesce P. Comparison of bone-level and tissue-level implants: a pilot study with a histologic analysis and a 4-year follow-up. *Int J Periodontics Restor Dent* 2022; **42:** 535–543. 10.11607/prd.4990 35830320

[13] Siebert C, Rieder D, Eggert J, Wichmann M G, Heckmann S M. Long-term esthetic outcome of tissue-level and bone-level implants in the anterior maxilla. *Int J Oral Maxillofac Implants* 2018; **33:** 905–912. 10.11607/jomi.6419 30025008

[14] Canullo L, Menini M, Bagnasco F, Di Tullio N, Pesce P. Tissue-level versus bone-level single implants in the anterior area rehabilitated with feather-edge crowns on conical implant abutments: an up to 5-year retrospective study. *J Prosthet Dent* 2022; **128:** 936–941. 10.1016/j.prosdent.2021.01.031 33715833

[15] Banihashem Rad S A, Forouzanfar A, Banihashemrad S A. Comparison of interdental papillae around single implants in the tissue-level (TL) and bone-level (BL) implants: a clinical trial. *J Adv Periodontol Implant Dent* 2020; **12:** 30–36. 10.34172/japid.2020.007 PMC9327465 35919306

[16] Garaicoa-Pazmino C, Mendonca G, Ou A *et al*. Impact of mucosal phenotype on marginal bone levels around tissue level implants: a prospective controlled trial. *J Periodontol* 2021; **92:** 771–783. 10.1002/JPER.20-0458 33107977

[17] Lazzara R J, Porter S S. Platform switching: a new concept in implant dentistry for controlling postrestorative crestal bone levels. *Int J Periodontics Restor Dent* 2006; **26:** 9–17. 16515092

[18] Hsu Y T, Lin G H, Wang H L. Effects of platform-switching on peri-implant soft and hard tissue outcomes: a systematic review and meta-analysis. *Int J Oral Maxillofac Implants* 2017; **32:** 9–24. 10.11607/jomi.5140 28095526

[19] Piattelli A, Vrespa G, Petrone G, Iezzi G, Annibali S, Scarano A. Role of the microgap between implant and abutment: a retrospective histologic evaluation in monkeys. *J Periodontol* 2003; **74:** 346–352. 10.1902/jop.2003.74.3.346 12710754

[20] Vervaeke S, Collaert B, Cosyn J, De Bruyn H. A 9-year prospective case series using multivariate analyses to identify predictors of early and late peri-implant bone loss. *Clin Implant Dent Relat Res* 2016; **18:** 30–39. 10.1111/cid.12255 24995626

[21] Lee B A, Kim B H, Kweon H H I, Kim Y T. The prosthetic abutment height can affect marginal bone loss around dental implants. *Clin Implant Dent Relat Res* 2018; **20:** 799–805. 10.1111/cid.12648 30048041

[22] Blanco J, Pico A, Caneiro L, Novoa L, Batalla P, Martin-Lancharro P. Effect of abutment height on interproximal implant bone level in the early healing: a randomized clinical trial. *Clin Oral Implants Res* 2018; **29:** 108–117. 10.1111/clr.13108 29222809

[23] Hermann J S, Buser D, Schenk R K, Schoolfield J D, Cochran D L. Biologic Width around one- and two-piece titanium implants. *Clin Oral Implants Res* 2001; **12:** 559–571. 10.1034/j.1600-0501.2001.120603.x 11737099

[24] Mattheos N, Vergoullis I, Janda M, Miseli A. The implant supracrestal complex and its significance for long-term successful clinical outcomes. *Int J Prosthodont* 2021; **34:** 88–100. 10.11607/ijp.7201 33570524

[25] Galindo-Moreno P, Leon-Cano A, Monje A, Ortega-Oller I, O'Valle F, Catena A. Abutment height influences the effect of platform switching on peri-implant marginal bone loss. *Clin Oral Implants Res* 2016; **27:** 167–173. 10.1111/clr.12554 25678247

[26] Heitz-Mayfield L J A, Heitz F, Lang N P. Implant disease risk assessment IDRA – a tool for preventing peri-implant disease. *Clin Oral Implants Res* 2020; **31:** 397–403. 10.1111/clr.13585 32003037

[27] Derks J, Schaller D, Hakansson J, Wennstrom J L, Tomasi C, Berglundh T. Effectiveness of implant therapy analyzed in a Swedish population: prevalence of peri-implantitis. *J Dent Res* 2016; **95:** 43–49. 10.1177/0022034515608832 26701919

[28] Bilhan H, Erdogan O, Geckili O, Bilgin T. Comparison of marginal bone levels around tissue-level implants with platform-matched and bone-level implants with platform-switching connections: 1-year results of a prospective cohort study with a split-mouth design. *Int J Oral Maxillofac Implants* 2021; **36:** 945–951. 10.11607/jomi.8891 34698721

[29] Lago L, da Silva L, Martinez-Silva I, Rilo B. Radiographic assessment of crestal bone loss in tissue-level implants restored by platform matching compared with bone-level implants restored by platform switching: a randomized, controlled, split-mouth trial with 3-year follow-up. *Int J Oral Maxillofac Implants* 2019; **34:** 179–186. 10.11607/jomi.6954 30282088

[30] Rodríguez X, Navajas A, Vela X, Fortuño A, Jimenez J, Nevins M. Arrangement of peri-implant connective tissue fibers around platform-switching implants with conical abutments and its relationship to the underlying bone: a human histologic study. *Int J Periodontics Restor Dent* 2016; **36:** 533–540. 10.11607/prd.2580 27333011

[31] Paul S J, Nesic D. Influence of the transmucosal surface of dental implants on the soft tissue attachment level and marginal bone loss in preclinical studies: a systematic review. *Int J Oral Maxillofac Implants* 2024; **39:** 173–183. 10.11607/jomi.10398 38416011

[32] Furuhashi A, Ayukawa Y, Atsuta I, Rakhmatia Y D, Koyano K. Soft tissue interface with various kinds of implant abutment materials. *J Clin Med* 2021; DOI: 10.3390/jcm10112386. 10.3390/jcm10112386 PMC8199343 34071480

[33] Calatrava J, Sanz-Sanchez I, Molina A, Bollain J, Martin C, Sanz M. Effect of one-time placement of the definitive abutment versus multiple healing abutment disconnections and reconnections during the prosthetic phase on radiographic and clinical outcomes: a 12-month randomized clinical trial. *Clin Implant Dent Relat Res* 2024; **26:** 998–1011. 10.1111/cid.13361 39011816

[34] Rathi P, Kolte R A, Kolte A P. Peri-implant tissue health in bone-level implants with repeated abutment replacements versus tissue- level implants with a definitive abutment: a randomized controlled trial. *Int J Periodontics Restor Dent* 2022; **42:** 33–42. 10.11607/prd.4792 35353086

[35] Quah B, Al Yousef A S, Lai C W M, Wong R C W, Chew R J J. Impact of implant design on outcomes of simultaneous guided bone regeneration for dehiscence defects: a systematic review and meta-analysis. *J Evid Based Dent Pract* 2024; DOI: 10.1016/j.jebdp.2024.102033. 10.1016/j.jebdp.2024.102033 39631971

[36] Puisys A, Auzbikaviciute V, Vindasiute-Narbute E, Razukevicius D, Linkevicius R, Linkevicius L. Influence of regular cover screw or 2 mm healing abutment on bone gain after conventional GBR: A randomised controlled clinical trial. 2019. Available at https://botiss.com/product/postereao2019-influence-of-regular-cover-screw-or-2mm-healing-abutment-on-bone-gain-after-conventional-gbr-a-randomized-controlled-clinical-trial-2/ (accessed 1 September 2026).

[37] Shen X, Yang S, Xu Y, Qi W, He F. Marginal bone loss of tissue- or bone-level implants after simultaneous guided bone regeneration in the posterior mandibular region: a retrospective cohort study. *Clin Implant Dent Relat Res* 2023; **25:** 68–76. 10.1111/cid.13144 36239210

[38] Chiapasco M, Casentini P, Zaniboni M. Implants in reconstructed bone: a comparative study on the outcome of Straumann tissue level and bone level implants placed in vertically deficient alveolar ridges treated by means of autogenous onlay bone grafts. *Clin Implant Dent Relat Res* 2014; **16:** 32–50. 10.1111/j.1708-8208.2012.00457.x 22494433

[39] Shahdad S, Makdissi J, Gambôa A. Relationship between facial bone dimensions, orofacial implant position, and esthetic outcomes of single-tooth implants. *Int J Prosthodont* 2023; **36:** 668–673. 10.11607/ijp.8052 38109386

[40] Ng E, Tay J R H, Mattheos N, Bostanci N, Belibasakis G N, Seneviratne C J. A mapping review of the pathogenesis of peri-implantitis: the biofilm-mediated inflammation and bone dysregulation (BIND) hypothesis. *Cells* 2024; DOI: 10.3390/cells13040315. 10.3390/cells13040315 PMC10886485 38391928

[41] Strauss F J, Park J Y, Lee J S *et al*. Wide restorative emergence angle increases marginal bone loss and impairs integrity of the junctional epithelium of the implant supracrestal complex: a preclinical study. *J Clin Periodontol* 2024; **51:**1677–1687. 10.1111/jcpe.14070 PMC11651719 39385502

[42] Yi Y, Koo K T, Schwarz F, Ben Amara H, Heo S J. Association of prosthetic features and peri-implantitis: a cross-sectional study. *J Clin Periodontol* 2020; **47:** 392–403. 10.1111/jcpe.13251 31912511

[43] Katafuchi M, Weinstein B F, Leroux B G, Chen Y W, Daubert D M. Restoration contour is a risk indicator for peri-implantitis: a cross-sectional radiographic analysis. *J Clin Periodontol* 2018; **45:** 225–232. 10.1111/jcpe.12829 28985447

[44] Chochlidakis K, Fraser D, Lampraki E *et al*. Prosthesis survival rates and prosthetic complications of implant-supported fixed dental prostheses in partially edentulous patients. *J Prosthodont* 2020; **29:** 479–488. 10.1111/jopr.13185 32364656

[45] Palma-Carrio C, Macconi A, Rubert-Aparici A, Vidal-Peiro P, Menendez-Nieto I, Blaya-Tarraga J A. Prosthetic complications of single screw-retained implant-supported metal-ceramic fixed prostheses: a retrospective observational study. *Int J Dent* 2024; DOI: 10.1155/2024/9242928. 10.1155/2024/9242928 PMC11335423 39165325

[46] Liu Y, Wang J. Influences of microgap and micromotion of implant-abutment interface on marginal bone loss around implant neck. *Arch Oral Biol* 2017; **83:** 153–160. 10.1016/j.archoralbio.2017.07.022 28780384

[47] Mattheos N, Larsson C, Ma L, Fokas G, Chronopoulos V, Janda M. Micromorphological differences of the implant-abutment junction and *in vitro* load testing for three different titanium abutments on Straumann tissue level implants. *Clin Oral Implants Res* 2017; **28:** 1523–1531. 10.1111/clr.13021 28397298

[48] Roccuzzo A, Jensen S S, Worsaae N, Gotfredsen K. Implant-supported 2-unit cantilevers compared with single crowns on adjacent implants: a comparative retrospective case series. *J Prosthet Dent* 2020; **123:** 717–723. 10.1016/j.prosdent.2019.04.024 31443973

[49] Malchiodi L, Cucchi A, Ghensi P, Consonni D, Nocini P F. Influence of crown-implant ratio on implant success rates and crestal bone levels: a 36-month follow-up prospective study. *Clin Oral Implants Res* 2014; **25:** 240–251. 10.1111/clr.12105 23402530

[50] Meijer H J A, Boven C, Delli K, Raghoebar G M. Is there an effect of crown-to-implant ratio on implant treatment outcomes? A systematic review. *Clin Oral Implants Res* 2018; **29:** 243–252. 10.1111/clr.13338 PMC6221159 30306696

[51] Thoma D S, Haas R, Sporniak-Tutak K *et al*. Randomized controlled multi-centre study comparing shorter dental implants (6 mm) to longer dental implants (11–15 mm) in combination with sinus floor elevation procedures: 10-year data. *J Clin Periodontol* 2024; **51:** 499–509. 10.1111/jcpe.13954 38296249

[52] Ravidà A, Serroni M, Borgnakke W S *et al*. Short (≤6 mm) compared with ≥10 mm dental implants in different clinical scenarios: a systematic review of randomized clinical trials with meta-analysis, trial sequential analysis and quality of evidence grading. *J Clin Periodontol* 2024; **51:** 936–965. 10.1111/jcpe.13981 38764386

[53] Lombardi T, Rapani A, Ezeddine F *et al*. Clinical outcomes of bone-level and tissue-level short implants placed in posterior maxilla: a case-control study. *Clin Implant Dent Relat Res* 2025; **27:** e13428. 10.1111/cid.13428 PMC11798899 39676168

[54] Gonzalez-Martin O, Lee E, Weisgold A, Veltri M, Su H. Contour management of implant restorations for optimal emergence profiles: guidelines for immediate and delayed provisional restorations. *Int J Periodontics Restor Dent* 2020; **40:** 61–70. 10.11607/prd.4422 31815974

[55] Chappuis V, Martin W. *Vol 10 ITI Treatment Guide*. Berlin: Quintessence Publishing, 2017.

[56] Buser D, Martin W, Belser U C. Optimizing esthetics for implant restorations in the anterior maxilla: anatomic and surgical considerations. *Int J Oral Maxillofac Implants* 2004; **19:** 43–61. 15635945

[57] Flügge T, Kramer J, Nelson K, Nahles S, Kernen F. Digital implantology – a review of virtual planning software for guided implant surgery. Part II: prosthetic set-up and virtual implant planning. *BMC Oral Health* 2022; DOI: 10.1186/s12903-022-02057-w. 10.1186/s12903-022-02057-w PMC8802526 35094677

[58] Vela X, Mendez V, Rodriguez X, Segala M, Tarnow D P. Crestal bone changes on platform-switched implants and adjacent teeth when the tooth-implant distance is less than 1.5 mm. *Int J Periodontics Restor Dent* 2012; **32:** 149–155. 22292143

[59] Tarnow D P, Cho S C, Wallace S S. The effect of inter-implant distance on the height of inter-implant bone crest. *J Periodontol* 2000; **71:** 546–549. 10.1902/jop.2000.71.4.546 10807116

[60] Rivara F, Macaluso G M, Toffoli A, Calciolari E, Goldoni M, Lumetti S. The effect of a 2-mm inter-implant distance on esthetic outcomes in immediately non-occlusally loaded platform shifted implants in healed ridges: 12-month results of a randomized clinical trial. *Clin Implant Dent Relat Res* 2020; **22:** 486–496. 10.1111/cid.12926 32633040

[61] Araujo M G, Silva C O, Misawa M, Sukekava F. Alveolar socket healing: what can we learn?. *Periodontol 2000* 2015; **68:** 122–134. 10.1111/prd.12082 25867983

[62] Hämmerle C H, Chen S T, Wilson Jr T G. Consensus statements and recommended clinical procedures regarding the placement of implants in extraction sockets. *Int J Oral Maxillofac Implants* 2004; **19:** 26–28. 15635943

[63] Trombelli L, Farina R, Marzola A, Bozzi L, Liljenberg B, Lindhe J. Modeling and remodeling of human extraction sockets. *J Clin Periodontol* 2008; **35:** 630–639. 10.1111/j.1600-051X.2008.01246.x 18498382

[64] Chan D, Pelekos G, Ho D, Cortellini P, Tonetti M S. The depth of the implant mucosal tunnel modifies the development and resolution of experimental peri-implant mucositis: a case-control study. *J Clin Periodontol* 2019; **46:** 248–255. 10.1111/jcpe.13066 PMC6593437 30638273

